# Physiological Mechanisms of Eccentric Contraction and Its Applications: A Role for the Giant Titin Protein

**DOI:** 10.3389/fphys.2017.00070

**Published:** 2017-02-09

**Authors:** Anthony L. Hessel, Stan L. Lindstedt, Kiisa C. Nishikawa

**Affiliations:** Department of Biological Sciences, Center for Bioengineering Innovation, Northern Arizona UniversityFlagstaff, AZ, USA

**Keywords:** giant sarcomeric proteins, muscle atrophy, muscle intrinsic properties, space travel, sports injury rehabilitation, titin/connectin, winding filament hypothesis

## Abstract

When active muscles are stretched, our understanding of muscle function is stretched as well. Our understanding of the molecular mechanisms of concentric contraction has advanced considerably since the advent of the sliding filament theory, whereas mechanisms for increased force production during eccentric contraction are only now becoming clearer. Eccentric contractions play an important role in everyday human movements, including mobility, stability, and muscle strength. Shortly after the sliding filament theory of muscle contraction was introduced, there was a reluctant recognition that muscle behaved as if it contained an “elastic” filament. Jean Hanson and Hugh Huxley referred to this structure as the “S-filament,” though their concept gained little traction. This additional filament, the giant titin protein, was identified several decades later, and its roles in muscle contraction are still being discovered. Recent research has demonstrated that, like activation of thin filaments by calcium, titin is also activated in muscle sarcomeres by mechanisms only now being elucidated. The *mdm* mutation in mice appears to prevent activation of titin, and is a promising model system for investigating mechanisms of titin activation. Titin stiffness appears to increase with muscle force production, providing a mechanism that explains two fundamental properties of eccentric contractions: their high force and low energetic cost. The high force and low energy cost of eccentric contractions makes them particularly well suited for athletic training and rehabilitation. Eccentric exercise is commonly prescribed for treatment of a variety of conditions including sarcopenia, osteoporosis, and tendinosis. Use of eccentric exercise in rehabilitation and athletic training has exploded to include treatment for the elderly, as well as muscle and bone density maintenance for astronauts during long-term space travel. For exercise intolerance and many types of sports injuries, experimental evidence suggests that interventions involving eccentric exercise are demonstrably superior to conventional concentric interventions. Future work promises to advance our understanding of the molecular mechanisms that confer high force and low energy cost to eccentric contraction, as well as signaling mechanisms responsible for the beneficial effects of eccentric exercise in athletic training and rehabilitation.

## Historical perspective

Nearly a century ago, critical experiments conducted largely by A. V. Hill and his students (Hill, 1922, 1938), defined the relationships among muscle force, velocity and power. Remarkably, these initial experiments were done with minimal technology, using little more than a stimulator, smoked kymograph drum, and several weights. Hence, these early experiments were limited to constant-length “isometric” and constant force “isotonic” muscle contractions. For decades, textbooks classified muscle use during movement and activity as fitting into one of these categories, isometric or isotonic. The principles and properties of isometric and isotonic muscle contractions seemed to adequately describe muscle force production *in vivo*. Effectively, the measurements had defined the perception of how muscles operate.

However, when the magnitude of a force applied to a muscle exceeds that produced by the muscle, the muscle will lengthen as work is done on it (often called “negative work”). The history of investigations into lengthening, or eccentric, muscle contraction is anything but straight forward. Even the term “eccentric” (which makes little sense, see (Faulkner, 2003) was first introduced as “excentric” by Asmussen (Asmussen, 1953). The original spelling is more appropriate as it combines the prefix ex-, “from or away,” with centric, “center,” hence a muscle “contraction” that moves away from the muscle's center. While A. V. Hill acknowledged that active muscles are often stretched (Hill, 1926), there was such a dearth of understanding of lengthening contractions that, 50 years after Hill's discoveries, the late bioengineer Tom McMahon referred to lengthening muscle contractions as the “dark side of the force–velocity curve” (Lindstedt et al., 2001).

### Low cost and high force of eccentric contractions

The observation that force generation in a muscle during active stretch was greater than that of a contracting muscle was first made by Fick (1882), and the magnitude of the extra force was later quantified by Katz (1939). These observation were supplemented by the results of A. V. Hill, who observed that the muscle being stretched also released much less heat (energy) (Hill, 1960). The functional significance of both of these properties of lengthening muscle contractions was demonstrated in a clever experiment by three of Hill's students: Bud Abbott, Brenda Bigland, and Murdoch Ritchie (Abbott et al., 1952). By attaching two stationary bikes back-to-back, one cyclist pedaled forward and the other resisted this forward motion by braking the backward-moving pedals. The much smaller Bigland resisting the pedals was easily able to equal the power output of the much larger Richie, and to do so with a tiny fraction of the oxygen consumption. For an outstanding review of this experiment, see (Elmer and LaStayo, 2014).

## Physiological mechanisms of eccentric contraction

Both “high force” and “low cost” (Ortega et al., 2015) are now well-established properties of eccentric contractions, demonstrable during movement (Seiberl et al., 2013, 2015) and in response to electrical stimulation (Lee and Herzog, 2002). Furthermore, these properties are demonstrable in isolated muscle preparations including intact muscles (Abbott and Aubert, 1952), single muscle fibers (Edman et al., 1982), single myofibrils (Joumaa et al., 2008a), and even single isolated sarcomeres (Leonard et al., 2010; Minozzo and Lira, 2013). Yet, current muscle models have difficulty explaining the increased force and reduced energy cost of eccentric contractions (Campbell and Campbell, 2011; Minozzo and Lira, 2013; Herzog, 2014a).

### Physiological mechanisms of increased force during and after active stretch

During an eccentric contraction, when an active muscle, fiber or myofibril is actively stretched, its force increases substantially during the stretch. Some of this extra force that develops during stretch decays when stretching stops, but the force that remains after stretching stops is substantially higher than the isometric force at the stretched length. This residual force (Edman et al., 1976) persists until the muscle is returned to its initial length or is deactivated. Cross-bridge models (Huxley, 1957; Huxley and Simmons, 1971) account for the increased force during stretch by assuming that cross-bridges are elastic and store energy when stretched to ~10 nm. Various additions to (Huxley's, 1957) two-state cross-bridge model have been proposed to account for the increased force during stretch, including rapid detachment and reattachment of cross bridges (Lombardi and Piazzesi, 1990) and tension-dependent rearrangement of the thick filaments (Linari et al., 2015).

Whereas these studies argue that cross-bridges alone may explain the magnitude of force enhancement, increasing evidence suggests that titin may also be involved (Linari et al., 2003; Pinniger et al., 2006; Roots et al., 2007). Cross-bridges alone cannot account for energy absorption during active stretch (Linari et al., 2003). Using a thermopile device in single fibers of frog tibialis anterior muscles, Linari et al. (2003) found that cross-bridge elasticity could only account for ~12% of the measured energy absorption during stretch. Even after accounting for tendon, thick and thin filament, and passive titin elasticity, only ~34% of the absorbed energy could be explained (Linari et al., 2003). Pinniger et al. (2006) and Roots et al. (2007) suggest that the P2 transition in the tension rise during active stretch that occurs at ~18 nm per half sarcomere (~1.3% of L_0_), is due to cross-bridges detaching from actin. They propose that the extra tension that remains after stretch to lengths >18 nm per half sarcomere is due to titin. Because the operating length of muscles are generally much larger than 1.1 L_0_, ~10% L_0_, (Prado et al., 2005), most of the absorbed energy during stretch is stored in non-cross bridge structures, mainly titin.

Prior to the discovery of titin in the late 1970's (Maruyama, 1976), researchers naturally sought explanations for eccentric contraction in the cross-bridges themselves. To date, however, studies have demonstrated that titin stiffness increases in activated muscle prior to development of force (Bagni et al., 2002, 2004; Rassier et al., 2015; Cornachione et al., 2016). Studies have also demonstrated a role for titin in residual force enhancement after stretch, (Leonard and Herzog, 2010; Powers et al., 2014; Herzog et al., 2016), and have further shown that an increase in titin stiffness persists for several seconds after a stretched muscle has been deactivated (termed “passive force enhancement,” Lee et al., 2007; Joumaa et al., 2008b). Given the weight of this evidence, it is now more parsimonious to presume that not only cross-bridges, but also titin, contributes to dynamic force production during active stretch because titin demonstrably contributes to stiffness during all other phases of an eccentric contraction.

### Residual force enhancement after stretch

Several hypotheses have been suggested to explain the increased muscle force that persists following eccentric contraction (Herzog, 2014b), including increased cross-bridge force, non-uniformities in sarcomere length (Morgan, 1990, 1994) or half-sarcomere length (Campbell et al., 2011), and engagement of structural elastic elements upon muscle activation (Edman et al., 1982; Herzog and Leonard, 2002; Leonard and Herzog, 2010). Here, we briefly review the evidence for and against these mechanisms.

#### Increased force of cross-bridges

While force enhancement following active stretch has been repeatedly documented across a wide range of skeletal muscle preparations (Campbell and Campbell, 2011; Herzog, 2014a), there is less evidence to support an increase in cross-bridge force during eccentric contraction (Minozzo and Lira, 2013; Herzog, 2014a). Because it is technically impossible to measure cross-bridge forces directly in muscle sarcomeres, observed changes in cross-bridge forces must necessarily be inferred from indirect measurements. Thus, modification of the quantity of attached cross-bridges can only be concluded from an observed modification in stiffness relative to force (Herzog, 2014a). Comparing the muscle stiffness in isometric vs. eccentric states has yielded contradictory conclusions, with some studies finding increased cross-bridge stiffness as predicted (Herzog and Leonard, 2000; Linari et al., 2000; Rassier and Herzog, 2005), while others found no difference in stiffness (Julian and Morgan, 1979) or even a decrease in stiffness after stretch (Sugi and Tsuchiya, 1981; Tsuchiya and Sugi, 1988; Piazzesi et al., 1992). Based on these observations, it appears that an increase in the number of attached cross-bridges is unlikely to account for residual force enhancement. An increase in the force produced per cross-bridge appears unlikely in that it would require cross-bridges to remain attached to actin for minutes rather than fractions of a second (Herzog, 2014b) and to be stretched by more than 300% of their resting length (Pinniger et al., 2006; Herzog, 2014b).

#### Sarcomere length non-uniformity

Another hypothesis is that force enhancement following eccentric contraction may be explained by non-uniformities in the force and length of muscle sarcomeres (Morgan, 1990, 1994). This hypothesis resulted directly from the failure of cross-bridge models to account for force enhancement (Harry et al., 1990). The sarcomere length non-uniformity theory makes several predictions: sarcomere length variability will be greater following stretch than during isometric contraction; force enhancement will be restricted to the descending limb of the force-length curve; and the force following stretch must not exceed the maximum isometric force. Each of these predictions is contradicted by substantial existing evidence (Herzog, 2014a,b). Force enhancement, though small, occurs on the ascending limb of the force-length relationship (Sugi, 1972; Pun et al., 2010). When the length of every sarcomere in series is measured in single myofibrils, the distribution of sarcomere lengths has been found to be more uniform in the force-enhanced state after stretch than in isometric contractions at the stretched length (Joumaa et al., 2008a). Finally, the force following active stretch has been observed to exceed the isometric force at the stretched length (Leonard et al., 2010).

Length non-uniformities have also recently been reported among half-sarcomeres upon active stretch of a single sarcomere (Edman et al., 1982; Telley et al., 2006; Rassier, 2012). However, the magnitude and duration of these observed increases in force due to half-sarcomere length non-uniformities are not large enough to adequately account for residual force enhancement (Stoecker et al., 2009; Campbell et al., 2011; Herzog, 2014a). Models based on half-sarcomere length non-uniformities predict that non-uniformity will increase upon active stretch compared to isometric contractions (Campbell and Campbell, 2011), in contrast to the decrease in non-uniformity observed experimentally (Joumaa et al., 2008a). Thus, this explanation fails to account for observed patterns of force enhancement.

#### Recruitment of structural elastic structures upon activation

Another hypothesis has been proposed that, upon muscle activation, structural elastic elements within sarcomeres increase in stiffness, thus contributing to force enhancement (Campbell and Campbell, 2011; Minozzo and Lira, 2013; Herzog, 2014a). Edman et al. (1976) were among the first to suggest that force enhancement involved recruitment of viscoelastic structures, after observing that single muscle fibers shorten faster in the force-enhanced state. Edman and Tsuchiya (1996) came to the same conclusion using load-clamp and unloaded shortening tests. Herzog and Leonard (2002) further demonstrated that when cat soleus muscle is stretched, the enhanced force persisted for several seconds after active stretch, even when the stretched muscle was deactivated. The elevated “passive” tension that persists after deactivation accounts for some of the force enhancement following active stretch, the simplest interpretation being that a structural element must contribute to force enhancement (Joumaa et al., 2008a). Likewise, when muscle fibers are stretched, there is increase in static tension during the early stages of muscle activation (Bagni et al., 2002, 2004; Nocella et al., 2014; Rassier et al., 2015).

## A role for titin in active muscle

When the sliding filament theory was introduced in 1954 (Huxley and Niedergerke, 1954; Huxley and Hanson, 1954), only the thick and thin filaments, composed of myosin and actin, had been identified in muscle sarcomeres (Rall, 2014). Far from being instantly accepted, the sliding filament theory in fact met with significant resistance (Maruyama, 1995). Perhaps for that reason, Hanson and Huxley (1957) inference, that an additional elastic filament (they named this hypothetical filament the “S filament”) must be present within the sarcomere, appears to have been largely overlooked. Despite the theoretical necessity of these filaments for sarcomere integrity, and in the face of accumulating evidence from electron microscopy (Sjostrand, 1962; McNeill and Hoyle, 1967), even Huxley (1964) and Hanson (1968)—who first suggested the existence of these filaments—were skeptical of the evidence, and it would be several decades before their existence became widely accepted. This acceptance would eventually require biochemical isolation and identification of giant proteins (Maruyama, 1976), as well as localization within the sarcomere using immunohistochemistry (Fürst et al., 1988).

Evidence is mounting that titin, activated by Ca^2+^ influx, is Edman's structural elastic element, along with the thin filaments in muscle sarcomeres. Leonard and Herzog (2010) demonstrated that if single myofibrils are activated by Ca^2+^ at a sarcomere length of 2.4 μm and stretched to a length beyond the thick and thin filament overlap (sarcomere length >3.8 μm), the force of myofibrils increases more rapidly with stretch than it does in passive myofibrils (Leonard and Herzog, 2010). Further, when sarcomeres are stretched beyond overlap, it is impossible for cross-bridges *per se* to contribute directly to active force. Also during stretch beyond overlap, the depletion of troponin C does not impact myofibril force, suggesting that the mechanism must not be related to thin filament activation (Powers et al., 2014). Finally, there was no “yielding,” i.e., decreased tension with stretch consequent to material failure (see e.g., Wang et al., 1991) when the sarcomeres were slowly stretched to long sarcomere lengths, implying little or no unfolding of Ig domains (Granzier, 2010; Rassier, 2012; Minozzo and Lira, 2013). These observations taken together led Leonard and Herzog (2010) to speculate that titin may bind to actin when Ca^2+^ is present, decreasing its free length and increasing its stiffness (Herzog et al., 2016), in addition to relatively small direct effects of Ca^2+^ on titin stiffness (Labeit et al., 2003; Joumaa et al., 2008a).

## Titin structure and function

Titin's molecular structure is unmistakably that of an elastic protein (Maruyama, 1976). It is the largest known protein, with a molecular weight up to ~4.2 MDa (Bang et al., 2001). Despite (or perhaps because of) its enormous size, titin was unknown to the Huxleys and thus was not integrated into the sliding filament theory (Huxley and Niedergerke, 1954; Huxley and Hanson, 1954). Since the discovery of titin, it has been speculated to contribute to passive muscle tension (Linke et al., 1998) as well as sarcomere integrity (Horowits and Podolsky, 1987). Horowits et al. (1986) demonstrated that titin filaments transmit cross-bridge forces to the z-disk. They used radiation to break titin filaments and electron microscopy to show that damage to titin results in a reduction of active tension in skinned single fibers, as well as axial misalignment of thick filaments. Without titin filaments, force generation by the cross-bridges is highly inefficient because energy from cross-bridge interactions is lost to thick filament displacement at the expense of longitudinal tension (Horowits and Podolsky, 1987; Horowits et al., 1989).

**Figure 1:**
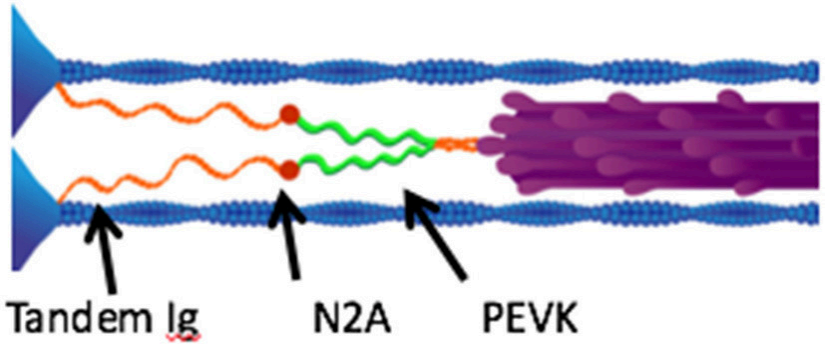
**Layout of titin and other muscle proteins in muscle sarcomeres**. Each titin molecule is bound to the thin filaments (blue) in the z-disk, and to the thick filaments (purple) in the A-band. The N2A segment (red) is located between the proximal tandem Ig segments (orange) and the PEVK segment (green). Reproduced with permission from Nishikawa et al. (2012).

Two serially linked spring elements make up titin's elastic I-band region: (1) tandem immunoglobulin (Ig) domains and (2) the PEVK segment (Figure 1: Gautel and Goulding, 1996). Passive stretch straightens the folded tandem Ig domains at relatively short sarcomere lengths, with minimal increase in tension. However, stretch to sarcomere lengths beyond the normal physiological range causes the PEVK segment to be stretched, resulting in a sharp increase in tension (Figure 1; Linke et al., 1998). Because these compliant and stiff segments are in series, titin-based passive tension is relatively small in comparison to active muscle tension. Thus, the conventional wisdom has not surprisingly been that titin, while elastic, is far too compliant to contribute to active muscle stiffness (Granzier and Labeit, 2004).

Despite this initial skepticism, increasing acceptance of the idea that titin plays a role in active muscle has led to a proliferation of hypotheses for physiological mechanisms, many of which involve interactions between titin and the thin filaments (Herzog et al., 2015). These include hypotheses that refolding of unfolded Ig domains may increase the speed of muscle shortening (Rivas-Pardo et al., 2016), that PEVK titin binds to thin filaments (Rode et al., 2009), and that the cross-bridges not only translate but also rotate the thin filaments, thereby winding titin upon them (Nishikawa et al., 2012).

To date, studies have demonstrated that: (1) titin stiffness increases in activated skeletal muscle prior to development of force (Bagni et al., 2002, 2004; Rassier et al., 2015); (2) titin stiffness contributes to residual force enhancement (Leonard and Herzog, 2010; Powers et al., 2014; Herzog et al., 2016); and (3) titin stiffness persists for several seconds after muscles have been deactivated (Lee et al., 2007; Joumaa et al., 2008b). The observed increase in titin-based stiffness in active skeletal muscles suggests that titin endows the sarcomere lattice with tunable stiffness, perhaps to more effectively transmit cross-bridge forces to the z-line (Horowits et al., 1986).

## The “winding filament” hypothesis

**Figure 2:**
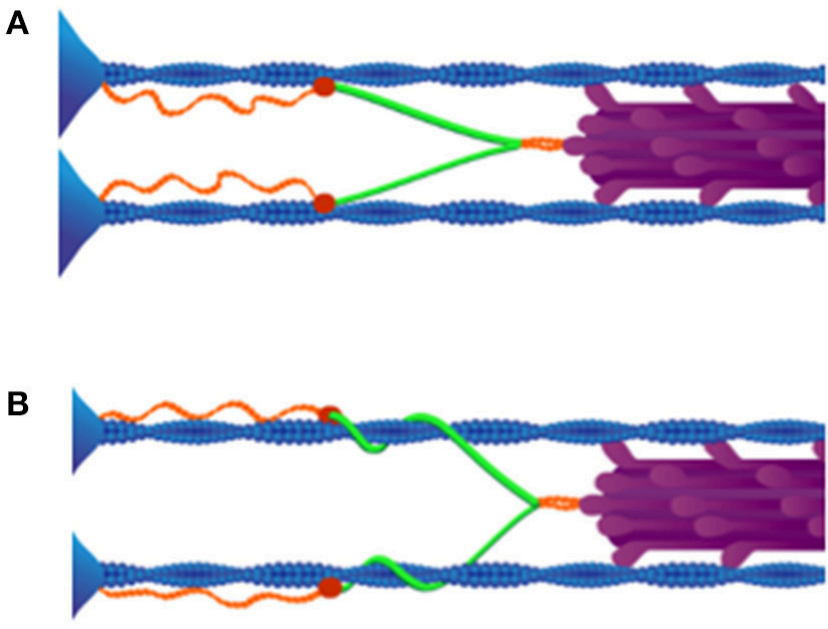
**Winding filament hypothesis. (A)** Upon Ca^2+^ influx, N2A titin (red) binds to thin filaments (blue). **(B)** Cross-bridges (purple) wind PEVK titin (green) on thin filaments in active muscles. As shown, all titins in the same half-sarcomere must wind in the same direction around actin filaments. Reproduced with permission from Nishikawa et al. (2012).

The winding filament hypothesis, proposed by Nishikawa et al. (2012), presents plausible molecular mechanisms for titin's role within active skeletal muscle sarcomeres in conjunction with both Ca^2+^ influx as well as cross-bridge cycling. The winding filament hypothesis proposes that following Ca^2+^ influx: (1) the N2A region of titin binds to actin; and (2) because the cross-bridges not only translate but also rotate the thin filaments, the PEVK segment of titin “winds” on the thin filaments during force development (Figure 2). Relatively little thin filament rotation (e.g., not <30° and no more than 200°, depending on sarcomere length) is required to account for observed muscle forces during eccentric contraction (Nishikawa et al., 2012). PEVK binding to actin (Bianco et al., 2007) is hypothesized to resist unwinding (Nishikawa et al., 2012).

### Titin-actin interactions

**Figure 3:**
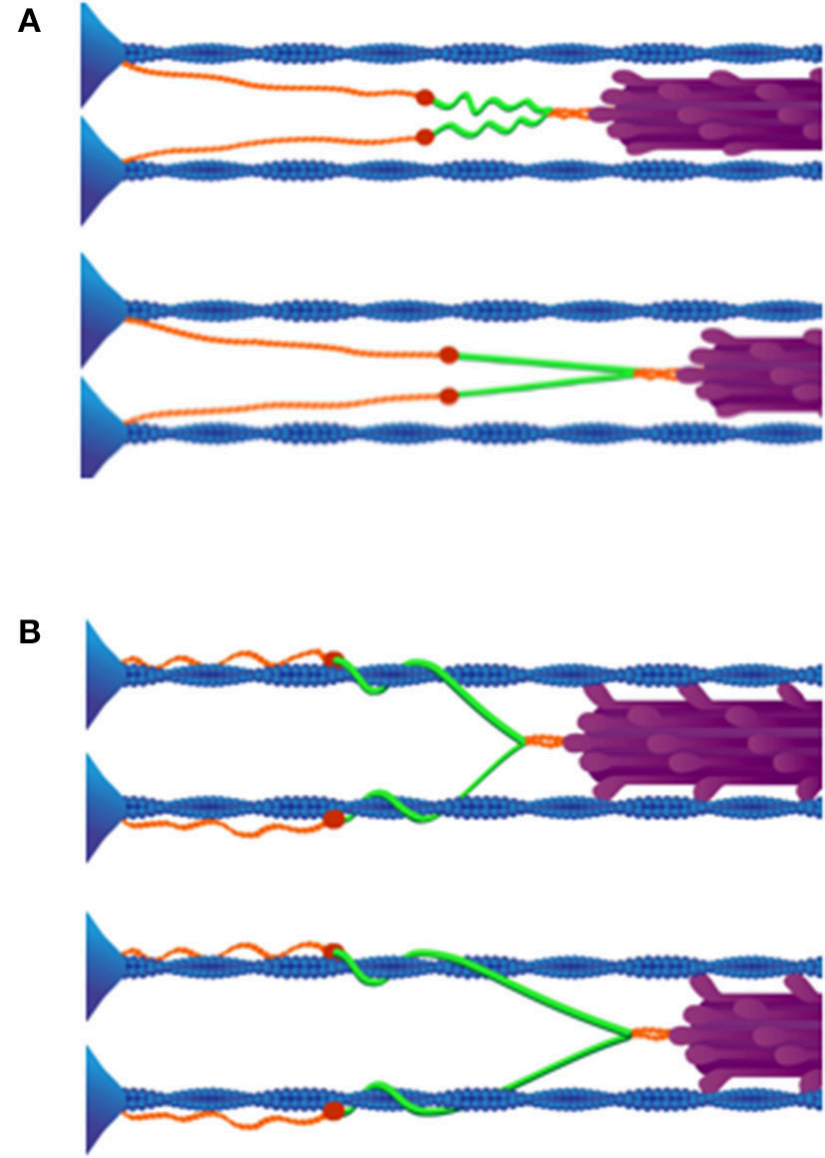
**(A)** Passive stretch of muscle sarcomeres. As a sarcomere is stretched beyond its slack length, the proximal tandem Ig segments unfold approximately to their contour length (above). After the proximal tandem Ig segments have reached their contour length, further stretching extends the PEVK segment (below). Adapted from Granzier and Labeit (2004). **(B)** Active stretch of muscle sarcomeres. Upon activation N2A titin binds to actin (above). Only the PEVK segment (green) extends when active muscle is stretched (below), due to binding of N2A to thin filaments. Reproduced with permission from Nishikawa et al. (2012).

The consequence of titin binding to actin during muscle activation, as described above, sufficiently accounts for the observed increase in titin-based stiffness upon activation in skeletal muscle myofibrils (Leonard and Herzog, 2010; Nishikawa et al., 2012; Herzog et al., 2016). The N2A region of titin is ideally located, separating the compliant tandem Ig domains from the stiff PEVK region; thus resulting in the modulation of titin stiffness via Ca^2+^-dependent binding to thin filaments (Nishikawa et al., 2012). When titin binds to actin in the N2A region, the low-force straightening of proximal tandem Ig domains in the I-band, seen during the passive stretch of myofibrils at slack length (Linke et al., 1998), is eliminated. As a consequence, when Ca^2+^-activated sarcomeres are stretched, only the stiff PEVK segment of titin will elongate, resulting in much higher forces (Figure 3).

While several studies have proposed that titin stiffness increases in response to Ca^2+^ (Labeit et al., 2003; Joumaa et al., 2008b), fewer studies have considered that in the presence of Ca^2+^, titin interacts with actin (Kellermayer and Granzier, 1996). The interaction they described occurred in an *in vitro* motility assay at a calcium concentration of 10^−6^ M; this is the critical concentration of calcium that results in passive force enhancement (Joumaa and Herzog, 2014). Immuno-antibody labeling of titin in rabbit psoas myofibrils demonstrates that titin movement differs significantly between passively and actively stretched myofibrils, suggesting that titin interacts with actin filaments in active skeletal muscle (Herzog et al., 2016). Tests are underway to determine whether and which domains of titin interact with actin in the presence of Ca^2+^. A mutant mouse with a deletion in the N2A region shows defects in titin activation (Powers et al., 2016).

### Titin-myosin interactions

Experiments by Edman et al. (1982) reinforced the necessity of including interactions between the cross-bridges and elastic elements in explaining the mechanism of residual force enhancement. In their experiments, active muscle fibers from frog tibialis anterior were shortened prior to stretch. Shortening an active muscle fiber prior to stretch should reduce or eliminate the extra force if the residual force enhancement were attributed to the activation of an elastic element. In contrast, they reported the persistence of the residual force enhancement despite the pre-shortening. They concluded that an elastic element, developing within the muscle during activation, is not slackened during shortening; an interaction with the cross-bridges is inferred to take up the slack.

Because the elastic region of titin is located at some distance from the thick filaments in the I-band (Gregorio et al., 1999), it would seem to preclude the possibility that titin interacts directly with myosin within the sarcomere. The concept that the thin filaments are rotated by cross-bridges and thereby winding titin upon actin, is one plausible means of titin-myosin interaction, although additional mechanisms have been proposed (Rode et al., 2009). The winding of titin on the thin filaments qualitatively resolves a mechanism linking cross-bridge force to titin force. This unique feature of the winding filament hypothesis is absent in other mechanisms that have been proposed.

The complexity of muscle sarcomeres means that it is technically difficult to test the winding hypothesis directly. Tests of this hypothesis are possible using transgenic mice that inducibly express fluorescently labeled titin, using electron tomography and holography, and using labeling of single myofibrils with fluorescent antibodies to titin. It is worth noting that there is now a considerable amount of indirect evidence that indeed cross-bridges both translate and rotate the thin filaments (see Nishikawa et al., 2012; Nishikawa, 2016), which is reviewed briefly below.

Importantly, isoforms of all known motor proteins track along a spiral path during their translation along helical microtubules and actin filaments, including dynein (Vale and Toyoshima, 1988; Can et al., 2014), kinesin (Brunnbauer et al., 2012), and non-muscle myosins (Ali et al., 2002; Sun et al., 2007). Additionally, heavy meromyosin has been reported to rotate actin filaments *in vitro* (Tanaka et al., 1992; Nishizaka et al., 1993; Sase et al., 1997). Finally, Nishizaka et al. (1993) reported that myosin heads create a right-handed torque on actin filaments along the length of their long axis, resulting in “super-coiling” of the actin double helix.

The purported rotation of actin by active myosin cross-bridges would necessarily result in the twisting of the thin filaments because actin filaments are anchored to the Z-disk, thereby reducing the helical pitch of the actin helix (Nishizaka et al., 1993). Using X-ray diffraction, changes in helical pitch of thin filaments have been observed in active muscle fibers (Bordas et al., 1999; Tsaturyan et al., 2005). These observations are consistent with the hypothesis that cross-bridge interactions with actin produce a right-handed rotation of thin filaments during isometric contraction and active shortening.

Thin filament rotation should also cause changes in Z-disk structure upon muscle activation. In the Z-disk, each thin filament is anchored to its adjacent thin filaments by four alpha-actinin “lanyards,” forming a small square pattern in relaxed sarcomeres when viewed in cross-section (Goldstein et al., 1990). When muscles either develop force isometrically or shorten isotonically, the Z-disk structure transforms from a small square to a basket-weave pattern (Goldstein et al., 1990). This orientation change of alpha-actinin is consistent with thin filament rotation.

### Predictions of the winding filament hypothesis

The unmodified sliding filament theory fails to explain residual force enhancement (Herzog, 2014a). However, by supplementing the sliding filament theory with a dynamic third titin filament that winds on actin, several perplexing observations of muscle physiology can be understood (Nishikawa et al., 2012). Thus, the winding filament hypothesis explains the observed force enhancement at low energy cost seen in eccentric contractions (Nishikawa et al., 2012, 2013; Joumaa and Herzog, 2013; Herzog, 2014a; Nishikawa, 2016). During a lengthening contraction, the work done on a muscle by stretching it, will extend titin, and in this process, elastic recoil energy is stored without the expenditure of ATP. The magnitude of stored energy will be a function of the stretch amplitude. The Ca^2+^-dependent binding of titin to the thin filaments explains why force increases much more during active than passive stretch. While stretching resting sarcomeres straightens the compliant N-terminal tandem Ig domains resulting in little tension, the stretch of active sarcomeres elongates the stiff PEVK segment (Monroy et al., 2012). The action of the cross bridges to stretch and wind PEVK on thin filaments provides an explanation for the recovery of residual force enhancement when an activated muscle fiber is shortened first, followed by stretch. This process also explains why residual force enhancement fails to increase as a linear function of initial sarcomere length upon activation, which would have to be the case with the engagement of a simple parallel elastic element during muscle activation (Edman et al., 1982).

## A role for giant sarcomeric proteins in other types of muscle

Titin is a relatively ancient protein, appearing in the common ancestor of bilateral animals, and orthologous giant proteins are found broadly across all animal taxa (Ohtsuka et al., 2011; Hanashima et al., 2012). Virtually all types of striated muscles found among animals also exhibit some form of length-dependence of force that resembles the force enhancement observed in skeletal muscles during eccentric contraction—from the Frank-Starling mechanism in cardiac muscle (Fukuda and Granzier, 2004) to catch tension in the muscles of invertebrates (Hooper et al., 2008). Recent results demonstrate a role for titin and other giant sarcomeric proteins in these phenomena as well (Yamada et al., 2001; Lindstedt and Nishikawa, 2017)

If titin is instrumental in eccentric contraction of skeletal muscle, how does the form and function of this elastic protein differ in cardiac muscle, which never contracts eccentrically? While cardiac and skeletal muscle have many properties in common, the nature of titin is not one of them (Lindstedt, 2016). Cardiac muscle only shortens when active. It never experiences isometric or eccentric contractions. In skeletal muscle, titin is very compliant when stretched passively. However, titin stiffness increases in the presence of Ca^2+^ in skeletal muscle, independent of muscle length. In contrast, titin stretch is always passive (during diastole) in cardiac muscle, and cardiac muscle titin is much stiffer passively than titin in skeletal muscle (Neagoe et al., 2003). Cardiac titin is functionally “designed” to be tuned to length changes during cardiac filling. Whereas, the more compliant titin isoforms of skeletal muscle can be tuned to any muscle length, the stiffer titin isoforms found only in cardiac muscle play a critical role in the Frank-Starling law of the heart (Fukuda and Granzier, 2004; Granzier and Labeit, 2004).

The phenomenon of catch has been mostly investigated in molluscan smooth muscles, however Wilson and Larimer (1968) found that “catchiness” is a general property of all invertebrate muscles. An elastic element develops upon muscle activation to create the catch state, which may persist for prolonged durations following deactivation; this elastic element displays modified stiffness during muscle shortening which results in the maintenance of force as the muscle shortens (Butler and Siegman, 2010). The elastic element of catch has been identified as twitchin, which is an invertebrate “mini-titin” (Funabara et al., 2007). Catch occurs when Ca^2+^ influx triggers the dephosphorylation of twitchin, followed immediately by the binding of twitchin to actin (Butler and Siegman, 2010). Although at least 26 different proteins change their phosphorylation state in catch (Hooper et al., 2008), Yamada et al. (2001) demonstrated that to produce the catch state *in vitro* only actin, myosin and dephosphorylated twitchin are necessary.

Both the structural similarities between twitchin and titin (Bullard et al., 2002), coupled with the functional similarities between catch and force enhancement following eccentric contraction, provide evidence supporting the likelihood that residual force enhancement of vertebrate skeletal muscle and invertebrate catch are evolutionary homologs (Lindstedt and Nishikawa, 2017). Although one would expect evolutionary divergence of the underlying biochemical mechanisms, unraveling the mechanisms of twitchin-based catch force may provide potentially rewarding insights into understanding residual force enhancement in vertebrate muscle.

## Applications of eccentric exercise for sports rehabilitation and space travel

**Figure 4:**
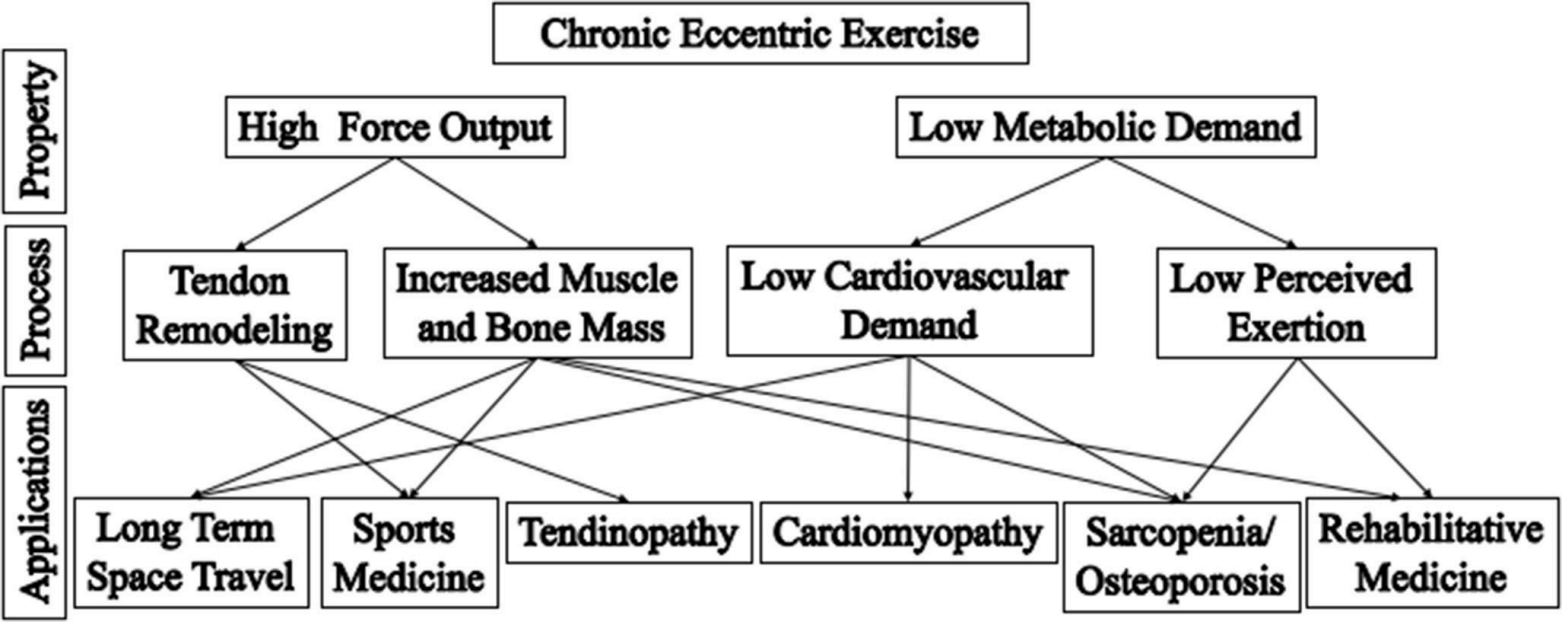
**Applications of eccentric training**. Eccentric contractions have the properties of high force output and low metabolic demand (top row). There properties allow the musculoskeletal system to be stressed in different ways, compared to conventional concentric exercise, leading to specific training benefits (middle row). These benefits are particularly appropriate for different applications (bottom row). For example, the low cardiac output and low perceived exertion that results from the low energetic cost of eccentric training is particular beneficial for rehabilitation of frail elderly or persons with cardiomyopathy. Adapted from Lindstedt et al. (2001).

**Figure 5:**
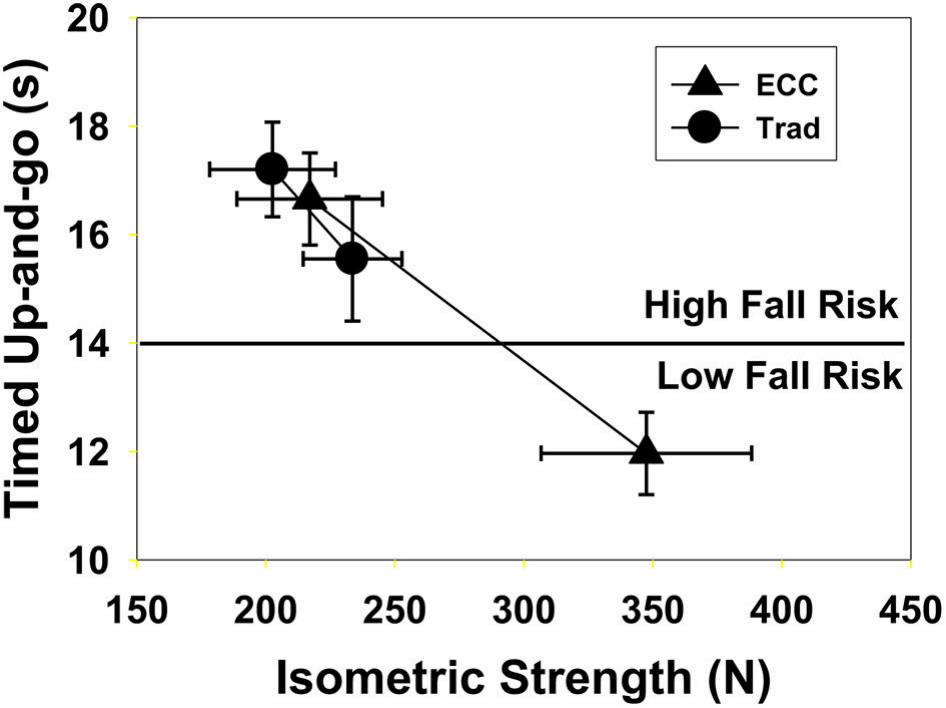
**Twenty-one frail subjects (mean age 80.2) were enrolled in an 11-week cardiopulmonary rehabilitation program**. Control subjects (10) used traditional resistance training with weights (Trad, circles) while the others (11) used an eccentric ergometer (ECC triangles). The Trad group had an insignificant increase in isometric strength (15%, *p* = 0.12) but a 1.7 s improvement in their timed up and go performance (*p* = 0.03). In contrast, the ECC subjects had a 60% increase in strength (p = 0.001) and performance on the timed up and go test improved by 4.7 s (*p* = 0.001); all but one subject changed from high to low fall risk. Reproduced with permission from LaStayo et al. (2003a).

A discussion of eccentric muscle contraction would be incomplete without some exploration of the unique clinical applications of eccentric contractions (Hoppeler, 2016). The high forces and low energy cost associated with eccentric contraction make eccentric exercise uniquely suited for a variety of applications (Figure 4). Eccentric training regimes range from low-to-moderate intensity exercises that are safe for exercise-intolerant and elderly persons (Figure 5) to high-intensity exercises that enhance athletic performance (Vogt and Hoppeler, 2014), prevent injury, and improve rehabilitation (LaStayo et al., 2003b; Roig et al., 2009).

Many reviews in the literature focus on eccentric exercise as a rehabilitation strategy for persons with exercise intolerance (e.g., cardiovascular or other conditions that limit training intensity; LaStayo et al., 2000; Reeves et al., 2009). For healthy subjects, exercise strategies—such as blood flow restriction with low force (Pope et al., 2013; Dankel et al., 2016)—may be just as effective as low to moderate eccentric exercises. However, the high-forces produced by muscles during eccentric contractions, and the high forces consequently exerted on muscles, bones and tendons, stimulate not only unique muscle hypertrophy and architectural adaptations (Franchi et al., 2014, 2015, 2016; Wisdom et al., 2015; Narici et al., 2016), but also bone mineralization and tendon remodeling (Reeves et al., 2009; Franchi et al., 2014; Wisdom et al., 2015).

High-intensity eccentric contractions are defined as those that produce forces above that possible during isometric or concentric contractions (English et al., 2014). To our knowledge, the only exercise strategy that effectively increases bone density and tendon remodeling in otherwise healthy adults is high-intensity eccentric exercise. We describe two unique clinical applications for high-intensity eccentric exercises: tendon injury repair and prevention of osteopenia and sarcopenia for astronauts in space.

### Eccentric contraction and muscle damage

In addition to the reluctant acceptance of titin as a muscle spring, a parallel history of eccentric contraction has been the widespread conviction that it must necessarily cause muscle damage (see e.g., Edwards et al., 1984). In retrospect, it is clear that any novel high-force muscle use can cause damage, but as eccentric contractions were both novel and usually high force, muscle damage was linked to eccentric contractions *per se*. In fact, any significant shift in an ordinary or repeated pattern of muscle use may result in muscle soreness and even inflammation, especially when either the magnitude or nature of muscle force production is varied. Muscle damage induced by an initial bout of novel exercise is such a common occurrence that an initial damaging bout of exercise has been proposed as a prerequisite for the initiation of muscle hypertrophy (Evans and Cannon, 1991; Smith et al., 1999; Hawke and Garry, 2001; Goldspink, 2003). The common symptoms after a bout of novel exercise include the delayed onset of pain, often accompanied by the presence of intracellular muscle enzymes or proteins in the serum, suggesting damaged fibers (Miles and Clarkson, 1994). The key functional change, which confirms impairment of muscle fibers and hence injury, is a decrease in muscular force production. Until recently, chronic eccentric training was seldom investigated experimentally, perhaps because of its strong association with muscle damage/injury.

A repeated pattern in science is that far less evidence is required to establish an idea as a “fact” than is required to dislodge an idea once established (the “sufficiency of proof” axiom). Once accepted, any observed cause-effect relationship becomes the paradigm within which future experiments are designed and interpreted. In fact, we have learned a great deal about how muscle responds to damage/injury through this valuable model of high-force, acute eccentric contractions (Clarkson and Hubal, 2002). However, it is essential to note that although eccentric contractions can and often do result in muscle damage/injury, they need not cause any measurable muscle damage or injury whatsoever (Flann et al., 2011). Unfortunately, the perceived link between eccentric contractions and muscle damage persists and likely delayed the use of chronic eccentric training in rehabilitation and athletic training.

Just as any novel task can result in muscle soreness, regular repetition of that task usually results in specific muscle adaptations that protect against damage or even soreness. When eccentric training begins initially with low forces and increases gradually in both force and duration over time, no injury occurs. Proposed explanations for the protective effect of repeated eccentric contractions (“the repeated-bout effect”) include changes in the recruitment of motor units with subsequent exposures to eccentric contractions, the elimination of weak areas of muscle fibers after an initial exercise bout, and the development of more resilient muscle structures (Stauber, 1989). What is certain, however, is that muscle injury, as defined by muscle soreness and/or elevated CK levels in the blood, is not a necessary prerequisite for these protective adaptations to occur (LaStayo et al., 2003a; Flann et al., 2011).

### Rehabilitation for sports injuries

A unique advantage of high-intensity eccentric training is shorter recovery times for some of the most common, long-lasting, and debilitating sports injuries (Ohberg and Alfredson, 2004; Ohberg et al., 2004; Frizziero et al., 2014). High-intensity eccentric training is an effective alternative to tendon remodeling surgery, with high success rates and shorter recovery times than conventional physical therapy (Alfredson et al., 1998; Ohberg et al., 2004; Alfredson, 2015).

Here, we discuss a well-studied example: chronic Achilles tendinosis. Chronic Achilles tendinosis is characterized by a long duration of Achilles tenderness with inflammation, abnormal tendon structure, and pain (Kvist, 1994; Ohberg et al., 2004) especially during activities, such as running. Pain leads to reduced physical activity and muscle atrophy (Ohberg and Alfredson, 2004; Galloway, 2013; Galloway et al., 2013). Conventional nonsurgical treatments, including physical therapy and rest, often fail to provide relief (Galloway et al., 2013; Alfredson, 2015). Surgery to repair the tendon is often prescribed, and may incapacitate athletes for months.

High-intensity eccentric training has shown promise as a treatment for chronic Achilles tendinosis. Alfredson et al. (1998) treated 15 athletes with severe Achilles tendinosis using 12 weeks of high-intensity eccentric exercise for the calf muscles. A group of 15 athletes previously treated with conventional therapies (e.g., rest, nonsteroidal anti-inflammatory drugs, and physical therapy) was used as a comparison group. After the intervention, all eccentrically trained athletes regained their pre-injury ability levels with decreased pain, while none of the comparison athletes showed marked improvements and all eventually underwent surgery (Alfredson et al., 1998). A follow up exam 4 years later with the high-intensity eccentric group demonstrated that tendon thickness had decreased and tendon structure improved in 12 of the 15 runners (Ohberg et al., 2004). Although it is unclear which musculoskeletal adaptations led to reduced pain, possible adaptations include decreased tendon stiffness (Morrissey et al., 2011), increased neovascularization (Ohberg and Alfredson, 2004), neuroplasticity (Rio et al., 2016), and increased “muscle shielding” (O'Neill et al., 2015). Muscle shielding is a new concept which proposes that eccentric training improves neuromuscular coordination and muscle strength, leading to reduced tendon loading, thereby improving tendon health (O'Neill et al., 2015).

### Space travel

A potentially useful application of eccentric exercise is for astronauts in space, again capitalizing on the high forces and low energy cost associated with eccentric exercise. As space travel increases in duration and extends farther beyond Earth's orbit, so do the attendant risks of living in microgravity (Ball, 2001; McPhee et al., 2009), including loss of bone density and muscle mass (Endo and Matsumoto, 2012; Ohshima and Matsumoto, 2012; Lloyd et al., 2014). Even with a mandatory exercise requirement of 2.5 h per day for astronauts, marked deterioration of muscle volume, muscle strength and bone density occurs after a few weeks of space travel, and continues to worsen with increased time spent in space (LeBlanc et al., 1995; Akima et al., 2000; Gopalakrishnan et al., 2010). In fact, bone mass is lost 10 times faster during space flight than in persons with osteoporosis (Ohshima and Matsumoto, 2012). These risks will only increase with missions to distant targets, such as Mars.

It seems that high-intensity eccentric contractions are the most effective strategy to maintain both muscle mass and bone density in otherwise healthy adults. For example, in a study by English et al. (2014), four groups of athletes (10 in each group, 40 total) performed eccentric supine leg press and calf press exercises with muscle loads equal to 0, 33, 66, 100, or 138% of their concentric one-repetition maximum force. After 8 weeks of training, both high-intensity eccentric training groups (100 and 138%) showed the largest increases in leg strength (a concentric one-repetition maximum leg press) and only the 138% group demonstrated an increase in bone mineral density (English et al., 2014). These results suggest that muscle forces above the concentric one—repetition maximum are necessary to stimulate bone growth in healthy individuals, making high-intensity eccentric training the exercise regimen of choice for astronauts during space travel.

Unfortunately, it is difficult and expensive to test new devices or exercise regimens under conditions of microgravity in a space craft due to the packed schedules and priorities of space agencies (Shiba et al., 2015). For example, Shiba et al. (2015) conducted an exercise program on the International Space Station (ISS) with their device, the Hybrid Training System. This 1.6 Kg electrical device stimulates elbow antagonist muscles to resist volitional contraction of the paired agonist muscle (instead of gravity). Although they found that the device reduced, but did not prevent, bone and muscle atrophy, the limitations of the study made the conclusions tentative. Only one astronaut participated in the study, and this astronaut only performed the exercises during the final 4-weeks of his 6-month mission aboard the ISS. The tools available to measure muscle and bone properties were also limited.

It is feasible to perform eccentric exercise in space? Currently, astronauts use an “advanced resistive exercise device” when exercising in space. The device uses vacuum cylinders and inertial flywheels to simulate gravity-resistive training (Loehr et al., 2011). Because this device is built for cyclical training that incorporates both concentric and eccentric contractions during exercise, modifications would be required to enable high force eccentric training. High force eccentric training could be achieved using the “advanced resistive exercise device” by following the 2-for-1 rule: use both limbs to perform work concentrically, and then shift to a single limb to perform the opposite motion eccentrically. However, it should be noted that incorporating a concentric phase in the workout will increase metabolic requirements. The astronauts' on-board food supply must accommodate demands resulting from any exercises intended to offset loss of muscle and bone. Therefore, a low energy cost exercise regime is highly desirable. To maximize metabolic savings, eccentric-only exercises, performed using dedicated eccentric ergometers, would be preferable. Although dedicated eccentric ergometers are expensive, they are safe and effective at increasing muscle mass for many populations (LaStayo et al., 2000, 2003b; Dibble et al., 2006; Gross et al., 2010).

## Conclusions

Early experiments conducted by A.V. Hill and his students demonstrated the unique high force and low-cost of eccentric muscle contractions in the first half of the twentieth century. Decades later, lengthening (eccentric) muscle contractions, though acknowledged, were rarely considered to be more than an oddity or perhaps a method for inducing muscle injury. Because eccentric contractions were not easily explained by conventional theories, several hypotheses were developed that attempted to explain eccentric contractions. Neither cross-bridge mechanisms nor sarcomere or half-sarcomere length non-uniformities can adequately account for the observations. Earlier suggestions that a structural elastic element might develop upon muscle activation were confirmed by recent findings that the stiffness of the giant titin protein increases upon activation in skeletal muscle. Incorporation of titin as a “third filament” in our concept of muscle sarcomeres has led to a profusion of new proposals for a role in active muscles. The “winding filament hypothesis” in particular explains several enigmatic muscle properties, including the high force and low cost of eccentric contractions. Ongoing experiments in single molecules, myofibrils, and intact muscles should soon provide the information necessary for critical tests of alternative hypotheses for titin-actin and titin-myosin interactions in skeletal muscle. New evidence suggests that giant sarcomeric proteins play an important role in the length dependence of force, not only in vertebrate skeletal muscles, but also in the Frank-Starling effect in cardiac muscle and catch tension in muscles of invertebrate animals. Recent research also demonstrates the benefits of eccentric training for numerous practical applications. These include rehabilitation for sports injuries, rehabilitation for persons who are intolerant of traditional exercise, elite athletic training, and training for astronauts during space travel when the risks of muscle and bone atrophy are high and a premium is placed on energy efficiency. High-intensity eccentric exercise results not only in muscle hypertrophy and increased bone mineralization, but also appears to improve tendon remodeling after injury. Elucidating the signaling mechanisms responsible for these beneficial effects of eccentric training should be a major priority for future research.

## Ethics statement

Studies involving human participants were performed in accordance with relevant institutional and national guidelines, with the approval of Northern Arizona University's Institutional Review Board for research involving human subjects, and with informed written consent from all human subjects involved in the study. Studies involving animals were carried out in accordance with the recommendations of the United States Department of Agriculture (USDA) Animal Welfare Act and Regulations (AWA), the Guide for the Care and Use of Laboratory Animals, Public Health Services Policy on Humane Care and Use of Laboratory Animals, Occupational Safety and Health Administration (OSHA), and Environmental Protection Agency (EPA) regulations, Northern Arizona University's Institutional Animal Care and Use Committee policies and procedures. The research was approved by Northern Arizona University's Institutional Animal Care and Use Committee.

## Author contributions

All authors contributed to the development, writing and editing of this paper. KN also provided final edits and collection of figures.

## Funding

This work was supported in part by the W. M. Keck Foundation to KN, the National Science Foundation (IOS 0732949, 1025806 and 1456868; IIP 1237878 and 1521231) to KN, and the Achievement Rewards for College Scientists Foundation to AH.

### Conflict of interest statement

One of the authors (SL) is a co-inventor of an ergometer licensed to Eccentron; BTE Technologies, Inc., Hanover, MD, USA. Neither SL nor any of the other authors have received any financial incentives (e.g., reimbursements, fees, royalties, funding, or salary) from the company stemming from the contents of this manuscript or any related published papers. The other authors declare that the research was conducted in the absence of any commercial or financial relationships that could be construed as a potential conflict of interest.
